# Depression care management for late-life depression in China primary care: Protocol for a randomized controlled trial

**DOI:** 10.1186/1745-6215-12-121

**Published:** 2011-05-13

**Authors:** Shulin Chen, Yeates Conwell, Baihua Xu, Helen Chiu, Xin Tu, Yan Ma

**Affiliations:** 1 Department of Psychology, 148 Tianmushan Road, Xixi Campus of Zhejiang University, Hangzhou, Zhejiang, 310028, China; 2 Department of Psychiatry, University of Rochester Medical Center, 300 Crittenden Blvd., Rochester, 14642, New York, USA; 3 Department of Psychiatry, Chinese University of Hong Kong, Hong Kong, China; 4 Department of Biostatistics and Computational Biology, University of Rochester, New York, USA; 5 Health Department of Shangcheng District, Hangzhou, Zhejiang, China

## Abstract

**Background:**

As a major public health issue in China and worldwide, late-life depression is associated with physical limitations, greater functional impairment, increased utilization and cost of health care, and suicide. Like other chronic diseases in elders such as hypertension and diabetes, depression is a chronic disease that the new National Health Policy of China indicates should be managed in primary care settings. Collaborative care, linking primary and mental health specialty care, has been shown to be effective for the treatment of late-life depression in primary care settings in Western countries. The primary aim of this project is to implement a depression care management (DCM) intervention, and examine its effectiveness on the depressive symptoms of older patients in Chinese primary care settings.

**Methods/Design:**

The trial is a multi-site, primary clinic based randomized controlled trial design in Hangzhou, China. Sixteen primary care clinics will be enrolled in and randomly assigned to deliver either DCM or care as usual (CAU) (8 clinics each) to 320 patients (aged ≥ 60 years) with major depression (20/clinic; n = 160 in each treatment condition). In the DCM arm, primary care physicians (PCPs) will prescribe 16 weeks of antidepressant medication according to the treatment guideline protocol. Care managers monitor the progress of treatment and side effects, educate patients/family, and facilitate communication between providers; psychiatrists will provide weekly group psychiatric consultation and CM supervision. Patients in both DCM and CAU arms will be assessed by clinical research coordinators at baseline, 4, 8, 12, 18, and 24 months. Depressive symptoms, functional status, treatment stigma and clients' satisfaction will be used to assess patients' outcomes; and clinic practices, attitudes/knowledge, and satisfaction will be providers' outcomes.

**Discussion:**

This will be the first trial of the effectiveness of a collaborative care intervention aiming to the management of late-life depression in China primary care. If effective, its finding will have relevance to policy makers who wish to scale up DCM treatments for late-life depression in national wide primary care across China.

**Study Registration:**

The DCM project is registered through the National Institutes of Health sponsored by clinical trials registry and has been assigned the identifier: NCT01287494

## Background

Depression is predicted to be the illness with the greatest negative impact and disease burden by the year 2020 [1]. Late-life depression affects approximately 8% to 16% of community-dwelling older adults in the U.S. [2], making it the most common mental health disorder of later life. Late-life depression is associated with physical limitations, greater functional impairment, increased utilization and cost of healthcare, and increased suicide and all-cause mortality [3]. Late-life depression also represents a major public health issue in China. In the city of Beijing, the 1-year prevalence of geriatric depression was 4.33% [4], and among patients in general hospitals in Shenyang the rate is 11% [5]. Rates of depression are even higher for patients with chronic diseases, (i.e., 78.9%) [6]. The total estimated cost of depression in China for 2002 is US$6.3 billion [7].

Currently, there is increased interest in targeting primary care settings for the treatment of depression, because most depressed individuals visit their primary care physicians (PCPs) during the course of their depressive episode [8]. Combinations of pharmacologic and psychosocial interventions currently available to PCPs have been shown to effectively treat patients with depression in primary care [9,10]. In Western countries, over one-half of all depression is treated in PC clinics [11]. The growth of available antidepressants, such as selective serotonin reuptake inhibitors (SSRIs), has made it safer and easier for PCPs to treat depression [12]. Given the low rates of older adults who seek specialty mental health services [13], it is crucial that PCPs place a high priority on recognizing and treating older patients with depression.

In 2006, the central government of China published guidelines on the development of community health services. They designated primary care a central component of the new strategy, with health education and promotion, outpatient evaluation, and case management of chronic disease and physical rehabilitation as the major responsibilities of primary care [14].

PC clinics in China have several advantages as a setting for mental health care delivery [15]. First, the convenience of PCC for elders: in most urban areas, residents can find a PC clinic within a half-mile radius around his/her house. Second, higher rates of insurance coverage: government health insurance covers a greater proportion of care when delivered through the PC system (90-100%) compared to care delivered in hospitals (70-100%). Third, PCC is responsible for the management of chronic disease: in each PCC, there are physicians and nurses for whom chronic disease management (e.g., diabetes) is a familiar activity. PC providers in these clinics are equipped to treat and manage chronic disease due to their training background and responsibilities *according the new National Health Policy. Meanwhile*, mental disorders are considered chronic diseases that also should be managed in primary care settings in the new National Health Policy of China [16]. Depression is a form of chronic disease due to its frequently relapsing and recurring course, making it a natural fit with the new emphasis on chronic disease management. Fourth, less stigma and fewer barriers to access: Epidemiological studies have shown that the Chinese had lower rates of late life depression compared with Westerns [17]. However, the over-65 age group has the highest rate of completed suicide, reaching 44.3-200 per 100 000 [18], which is four to five times higher than the Chinese general population; and depression is a major cause of suicide among older adults [19]. Chinese culture, stigma, and a tendency to express depression somatically in Chinese elders make them reluctant to seek care from mental health specialists [20,21]. Individuals may be more likely to seek treatment for mental health problems in a primary care setting [22], as less stigma is associated with primary care treatment compared to mental health care clinics in China, just as in the West [23].

Despite the potential role for primary care in the management of depression, low levels of detection and treatment of depression have been highlighted in primary care settings [24]. A range of patient, doctor and practice barriers contribute to the under-recognition and less than optimal management of depression in primary care settings: at the patient level, comorbidity and reluctance to speak about depressive symptoms; at the provider level, PCPs' lack of knowledge and skill; and at the practice level, lack of adequate consultation time and insufficient access to specialized mental health resources [25-27]. Up to 80% of patients with a diagnosable depressive or anxiety disorder present with somatic complaints, which may affect PCPs' ability to recognize and diagnose depression or anxiety [28]. Patients' limited awareness regarding depression and its frequent manifestation through nonspecific physical symptoms, especially in China is also an important barrier to accurate diagnosis of depression [27,29,30]. Depressed patients with higher stigmatized beliefs regarding mental health are less adherent to pharmacologic treatments [31,32]. For PCPs, knowledge and skills appear to influence the recognition and appropriate management of depression [33]. PCPs who demonstrate better conceptual understanding of mental illness also demonstrate greater diagnostic accuracy [34]. The specific beliefs and attitudes that PCPs hold toward depression also appear to impact upon their diagnosis and management of depression [35].

Based on the principles of the *Chronic Disease Management Model*, and derived from successful research conducted in patients with depression, the "collaborative care model (CCM)" for depression in adults and older adults has sufficient evidence for dissemination and implementation [36,37]. The CCM for depression is not a single intervention or set of interventions, but a principle-guided approach to the treatment and management of depression. Interventions that can be classified as CCM can range from simple telephone interventions to encourage compliance with medication [38] to complex interventions at the "systems level." The prototypical ingredients of CCM at the systems level include the following: 1) a multi-professional approach to patient care, 2) activated patients, 3) a structured patient management plan (e.g., recognition and assessment of depression, psychoeducation, treatment guidelines, monitoring of outcomes), 4) scheduled patient follow-up, and 5) enhanced inter-providers communications [24,39]. A variety of recent randomized controlled trials have demonstrated that when treatment guidelines (TG) are integrated into a practice with a multifaceted and longitudinal treatment approach, significant improvements in guideline-concordant care and patient outcomes result [40-48]. This research indicates that the three classes of professionals integral to CCM are 1) PCPs, 2) care managers, and 3) mental health specialists [49].

## Objectives

Based on chronic disease management theory and previous CCM studies in western countries, we propose to test a Depression Care Management (DCM) intervention, which includes TG to support PCPs management of depression in their older patients; primary care nurses as care managers (CM) to monitor the progress of treatment, support patient's adherence, educate patients/family and facilitate communication between providers; and psychiatrists to provide consultation and supervision of care managers. Using a randomized controlled design, we will examine whether the DCM is an effective treatment for patients with late life depression in urban China. The primary outcome would be the improvement of depressive symptoms of patients in primary care setting.

Our specific aims are:

1) To determine whether the DCM intervention results in improved outcomes compared with care as usual (CAU) at patient levels (e.g., greater reduction in depressive symptoms);

2) To determine whether the DCM intervention results in improved outcomes compared with care as usual (CAU) at provider level (e.g., greater adherence to quality indicators);

3) To compare DCM with CAU with regard to a range of outcomes in other pertinent domains, both at the provider (e.g., improvements in knowledge/attitudes) and patient (e.g., functioning, satisfaction) levels.

The DCM trial is a collaborative effort between institutions in China. The lead institution is the Department of Psychology Zhejiang University, whiles the partner organizations are the Department of Psychiatry University of Rochester Medicine Center, the Department of Psychiatry Chinese University of Hong Kong, the Department of Health in Hangzhou City, the Mental Health Center of Hangzhou City. The study protocol was approved by the Human Study Committee in Children Hospital of Zhejiang University (OHRP IRB #00003806).

## Methods/Design

Study design: Two-stage screening and randomized-controlled trial comparing collaborative depression care management (DCM) to care as usual (CAU) in the treatment of late life depression.

All older adult patients who receive care at participating clinics will be approached by clinic administrative personnel with the opportunity to participate in the first stage of depression screening (who will obtain verbal consent to release the patient's contact information to the study team). The first stage of screening involves completion of a depression screening tool (i.e., PHQ-9) in order to maximize efficiency of time allocation for study personnel. Only patients who score above the study-determined threshold on the PHQ-9 (see below) will be invited to participate in the second stage of screening (i.e., structured diagnostic clinical interview).

### Study location

The study will take place in 16 PCCs in Hangzhou City. In the urban area of Hangzhou City, there are 185 primary care clinics (PCCs) [50] providing services for 4.19 million residents (16% of them aged ≥ 60 in 2008) [51]. Each clinic: 1) has 1-2 doctors and 3-4 nurses, 2) is funded by the district government and supervised by a primary care center in this district, 3) does not include an inpatient service or university affiliation, 4) provides care for 8,000-12,000 residents. The routine service of each PCC involves provision of the following [52]: 1) outpatient medical care, **2) **preventive medicine, **3) **chronic disease management (currently only two diseases: hypertension and diabetes), 4) health education, 5) physical rehabilitation, 6) family planning.

Sixteen of the 30 PCCs in the Shangcheng District will be randomly selected and assigned to the DCM intervention condition or the control condition (i.e., CAU). a) Assignment will be by a remote computer-generated number sequence concealed from researchers. b) Included PCCs will be informed of their assignment to DCM or CAU after patients are enrolled in the study by research interviewers. c) The randomization will be at the clinic level (i.e., rather than patient level).

Study conditions: CAU and DCM

CAU: Current practice, when depression is detected by PCPs, involves suggesting to patients (or family members) that they consult a mental health institution for diagnosis and treatment. There is no direct referral/transfer mechanism between PCPs and mental health specialists. Occasionally PCPs prescribe antidepressants following mental health specialists' protocols on patients' medical records. To evaluate the potential effectiveness of the DCM intervention, information is needed on patients in the CAU as well as DCM. For ethical reasons PCPs in both groups will be informed of the screening results (i.e., depression diagnoses from the structured clinical interviews). In previous studies, identification of depression alone or usual referral has been shown to have little effect on patient outcomes [53-57]. This indicates that there is very low possibility that providing diagnostic data to CAU PCPs will diminish the hypothesized differences between the two groups. However, the provision of this diagnostic information does go beyond usual care practices, thus indicating that "enhanced care as usual" would be the most precise descriptor for our control condition. For ease of communication, however, we will refer to our control condition as CAU.

### DCM intervention

Treatment guidelines (TG): The Depression Management Toolkit [58] used in the Three-Component Model (TCM) for late life depression will be translated and adapted for use with Chinese PCPs. Provided to us by the MacArthur Initiative for Depression in Primary Care the toolkit includes recognition and diagnostic information, patient education materials, treatment information, and monitoring and follow-up information. In addition to translation, the adaptations in this study are the treatment guidelines and the management approach for treating depression. With the improvement of treatment and antidepressants in recent years, the adaptation of TG for late life depression is necessary. The Duke Somatic Treatment Algorithm for Geriatric Depression (STAGED) was designed for geriatric depression [59] and all drugs in the STAGED are now readily accessible in China. We will adapt the STAGED to just two stages: 1) 8 weeks treatment with sertraline, 2) another 8 weeks treatment augmentation with bupropion if patients fail to respond in the initial trial. For more complicated cases, the transfer to psychiatrists is indicated. We need to provide PCPs' with a simple, straightforward treatment protocol because they have essentially no mental health training. With the adaption and integration from the STAGED approach, we will utilize two of the treatment approaches in DCM: the "antidepressant medications" and the "mental health referral". Currently, the provision of psychological counseling by Chinese PCPs and nurses is not feasible due to their training background. Results from our preliminary studies indicate that only 12.7% of PCPs felt confident in the delivery of evidence-based psychotherapy. The medication treatment protocol is listed below:

PCPs allocated in the DCM intervention group will treat recruited patients with medication treatment for 16 weeks.

1). Initial trial: Patients with major depression will receive an 8-week initial trial of sertraline. The initial daily dose will 25 mg (a.m.) and increased a week later to 50 mg (a.m.). The dose can be increased to 100 mg/day at Week 3 and 150 mg/day at Week 6. Study physicians will have the option of changing the dosage by 25 mg increments if the patient reports difficulties with side effects. The maximum dosage allowed is 150 mg/day. Sertraline is efficacious in reducing depressive symptoms, safe, and well tolerated by patients with or without medical illness [60]. Given current practices in PCCs in China, it is not expected that patients will request alternative medications; however, should this issue arise, patient choice will be respected (when feasible) and this departure from the algorithm will be noted in clinical monitoring forms.

2). Second stage: If the initial trial produces a good response (50% decrease in score on PHQ-9 from baseline to Week 6), the sertraline treatment will last another 8-weeks. If patients fail to respond in the initial trial, patients will receive an 8-week trial of sertraline augmentation with bupropion with the psychiatrist's consultation. After unsuccessful treatment with an initial SSRI, bupropion provides a reasonable second-step choice for patients with depression [61]. We will not provide PCPs the option of switching to venlafaxine due to studies documenting risk for cardiovascular side effects for patients with late life depression using Venlafaxine [62-64].

3). Follow-up: Patients treated under the above protocol will be followed by care managers. During follow-up, the care manager will administer the PHQ-9, SSI, side effects scale, and record of adherence to recommendations (see Measures section below for descriptions). At 16 weeks, patients who are either asymptomatic or have minimal depressive symptomatology will receive continuation treatment, while patients with clinically significant depressive symptoms and signs (<30% decrease in score on HAMD from baseline to Week 16) will be referred for psychiatric consultation.

4). Continuation treatment: Patients who are asymptomatic or have minimal symptoms after 16 weeks treatment with Sertraline alone or augment with Bupropion will receive continuation treatment for 6 months and be followed by CRCs through telephone contact. The dosage of Sertraline or/and Bupropion will be the same as the above treatment phase. After the continuation treatment, patients will discontinue or continue their medications with psychiatric consultation.

Care managers (CM): In this study, primary care *nurses in the primary care clinics (PCCs) will be employed as care managers for these reasons: *1) there are not enough mental health specialists in Chinese primary care settings; 2) PCPs will not accept outside providers working with them on a regular basis; 3) there is a full-time nurse in all PCCs in the study location who is assigned to chronic disease (including hypertension and diabetes) management for older patients in primary care. Adding the depression management care to their existing responsibilities will facilitate implementation of the DCM, as doing so is easier relative to creating a new jobs in the primary care system; 4) nurses in PCCs are familiar with many older patients in the neighborhood, thus promoting higher acceptability to patients relative to case managers outside the community; 5) nurses' clinical knowledge regarding medication side effects, their ability to treat chronic disease, and their ability to establish rapport with PCPs are also advantages for care management; 6) care managers who can coordinate the care delivered by mental health specialists and PCPs-including nurses-will best be able to sustain their services, regardless of their specific clinical training [24]. Although the lack of specific knowledge and skills on late life depression representing a major barrier for the use of nurses as care managers, we will draw upon the care manager manualized training and toolkit for nurse in the TCM studies-that has demonstrated effectiveness [65-68]. After training, care managers will acquire skills to: 1) score the PHQ-9 to assess symptoms and severity of depression both initially and in follow-up contacts; 2) use focused questions to evaluate suicide risk; 3) use patient education materials to promote adherence to the management protocols; 4) Conduct initial and all subsequent care management calls; 5) complete CM reports to effectively communicate call outcomes and patient status to PCPs; 6) prepare forms for efficient care management supervision with the supervising psychiatrist. And the communication between the professionals and care managers in the DCM intervention will be facilitated by: 1) a weekly case conference including psychiatric consultants, 2) weekly case management supervision, 3) communication forms between providers, 4) patients' tracking forms for the intervention and its progress.

Psychiatrists: The clinical complexities of treating severe depressive episodes and episodes occurring simultaneously with various physical illnesses often lead PCPs and care managers to consult with mental health specialists. In our study, a psychiatrist will conduct weekly group supervision of care managers and PCPs in a 1-hour case conference (for each DCM clinic). The resources and backup they provide for the PCPs will allow the PCPs to be more confident in managing patients with late life depression. The supervision for PCPs and care managers has been demonstrated to be an important component determining the effectiveness of a CCM [69].

Two psychiatrists with over 10 years of clinical practice experience, will offer enhanced mental health support for PCPs and care managers. Psychiatrists will perform three major services: 1) supervising CM, 2) providing informal consultation to PCPs, 3) increasing the quantity and quality of mental health referral resources. The weekly 1-hour case conference for all PCPs and case managers in the DCM intervention group will be an approach for delivering these services. The conference will be held at PCCs.

### Participants' recruitment and intervention

Older Adult Research participants: All contacts with primary care doctors in 16 PCCs may be utilized for research. These are older adults (aged ≥ 60) who are living in the community and contact the primary care doctors in this community for their physical health care. Data collected by specialists at initial contact may come either from the older adults him/herself or from his/her family. Care managers and research personnel will collect data from the older adult him/herself. The inclusion and exclusion criterion are listed below:

Inclusion Criteria: 1) Age ≥ 60 years: The retirement age is 60 years old for most people in China, and they will be regarded as older people. 2) Community-dwelling residences: Subjects must be registered residents of the community, and thus also of the community's PCC. We will not recruit older patients in hospitals or other health institutes, and no temporary residents (e.g., migrants). Institutionalized patients are different from primary care patients on screening, diagnosis and treatment strategies. Less than 2% of older residents in the community are temporary; they are not eligible for routine care in the PCCs and it is very difficult to track their medical records. 3) Capable of independent communication: The ability of independent communication is necessary for the 1-2 hours interview and baseline in-depth assessment, it is also necessary for telephone contact and follow-up with the care managers. Hearing or vision problems are very common in older people. If subjects have vision problems but can hear and understand the conversation in person and by telephone, they will be included. If a subject has mild or moderate hearing problems but can communicate with hearing aids, they will also be included. 4) Mini-Mental State Examination (MMSE) score ≥18: As depression is associated with cognitive impairment, inclusion of patients with mild cognitive problems is important to this study. But if patients have moderate to severe cognitive impairment (as indicated by ≤17 on the MMSE) on the initial assessment, the reliability of self-reported information obtained from these patients would be expected to be low, and PCPs may have greater difficulty in treating the complicated case with comorbidity of depression and dementia.

Exclusion Criteria: 1) Incapable of giving written informed consent to this study: Patients who are unable to comprehend the purpose and procedures of the study, appreciate the risks and alternative, or agree to the study for irrational reasons will be considered unable to sign consent. We will test their capacity to consent by questioning their understanding of the consent form as presented by the research assistant prior to enrollment. 2) Acute high suicide risk at baseline assessment: If patients are found to be at acute high suicide risk based on the psychiatrist's assessment at intake, they need immediate intervention such as referral to mental health specialists or informing patients' relatives. They will be excluded from the study at that time. Patients assessed to be dangerously suicidal at later assessments after enrollment will be discontinued from the study (an outcome measure of interest), their providers notified, and their safety guaranteed. 3) Psychosis: We exclude patients with psychosis to ensure our ability to test the proposed hypotheses. Few older adults (3.4%) have heavy alcohol drinking in urban China; and the current rate of alcohol abuse in elderly is lower than 1% [70-72]. Therefore we will not exclude subjects on that basis.

### The flow of participants in the trial

Administrative staffs in each PCC administer the PHQ-9 screening program before patients' visit with doctors. In the allocated 16 PCCs, all patients who screen ≥10 on the PHQ-9 will be invited to participant in our study. After the verbal consent to release their contact information to the study team, patients' name and contact information will be provided to Clinical Research Coordinator (CRC) who will contact patients and invite them to participate in the second stage of screening, the structured diagnostic interview.

A CRC will initiate contact with recruited patients and introduce our study (aims, procedures, risks and benefits). The CRC will obtain informed consent for the diagnostic interview. After written informed consent is obtained, a psychiatrist who is blind to the study design will conduct a diagnostic interview and in-depth assessment of recruited patients. The interview will take place in patients' homes or in the PCCs' offices at the patients' discretion.

**Figure 1:**
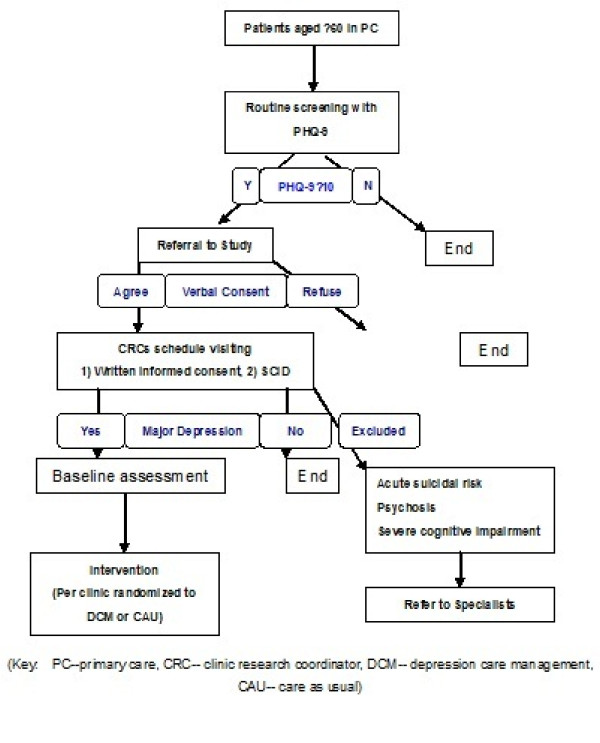
**Participants Recruitment Flowchart**. (Key: PC-primary care, CRC- clinic research coordinator, DCM- depression care management, CAU- care as usual)

After completion of the baseline diagnostic interview, eligible participants (i.e., those with major depression) who consent to participation will be assigned CAU or DCM based on which PCC they receive care from (*see *Figure 1). If patients present with acute suicide risk, psychosis, or severe cognitive impairment during the baseline assessment (i.e., exclusion criteria for the study), their PCP and family will be notified, and they will be given a referral for mental health specialists.

Every physician in the DCM intervention condition will receive a "paper case" a week before the training program (a training case adapted from the Depression Management Tool Kit in TCM studies). The 2.5-h training program chaired by the investigator will be conducted in each clinic. PCPs will be trained to use the depression medication algorithm based on the STAGED guidelines, provided with the skills needed for use of a depression diagnosis and response measure, and instructed in the use of communications forms and routines. Finally, physicians will be trained on how to fill the Clinical Record Form (CRF) for this study.

### D4. Measures

D4.a At the patient level: We will gather information about age, gender, education, marital status, living conditions and satisfaction with their economic status, as well as systematic information on suicidal ideation, psychopathology, medical health, cognitive function, quality of life and stigma and satisfaction for the treatment.

D4.b At the provider level: the objective of research measures at the provider level is to gather social-demographic data such as age, gender, education, marital status, clinical experience among physicians in the participating PCCs as well as systematic information on their attitudes/knowledge regarding depression and clinical practices with the treatment guidelines.

Almost all assessment instruments-both patient and provider level-are currently in use in China. The schedule of administration of these measures is summarized in Table 1.

**Table 1:** Summary table of data collection in DCM trial

Level	Assessment	Baseline	4 months	8 months	12 months	18 months	24 months
Patient	SDQ	√					

	SCID	√					

	HDRS	√	√	√	√	√	√

	SSI	√	√	√	√	√	√

	CAS	√	√	√	√	√	√

	SF-12	√	√	√	√	√	√

	MMSE	√			√		√

	CIRS	√			√		√

	TS	√	√	√	√		√

	CSQ-PCC	√	√	√	√		√

Provider	SDQ	√					

	DAQ	√	√	√	√	√	√

	PQ		√	√	√		

	CSQ-I		√	√	√	√	√

Key: ***SDQ***-Social Demographic Questionnaire; ***SCID***--Structured Clinical Interview for DSM-IV; ***HDRS***-24-item Hamilton depression scale; ***SSI***-Scale for Suicidal Ideation; ***CAS***-Clinical anxiety scale; ***SF-12***-12-item Short-Form Health Survey; ***MMSE***-Mini-Mental State Examination; ***CIRS***-Cumulative Illness Rating Scale; ***TS***-Stigma on Medication treatment for Depression; **CSQ-P-**Client Satisfaction Questionnaire for all patients in PCCs; ***DAQ***-Depression Attitude Questionnaire; ***PQ***-Practice Questionnaire, based on the Clinical Record Form and Medical Records in the authorized administration system; ***CSQ-I***-Client Satisfaction Questionnaire for all providers in DCM and CAU intervention.

### Outcomes assessment

#### Patient's level

1) Depression - The Mood Disorder Module of the Structured Clinical Interview for DSM-IV (SCID-MD), will be used to derive intake depressive diagnoses [73] and the Hamilton Rating Scale for Depression (HDRS, 17-item version) will be used to measure depressive symptom severity [74]. The Chinese version of SCID has good inter-rater reliability: the overall percentage agreement for principal diagnoses was 80%, which improved to 87% when only psychiatric patients were considered [75]. The reliability and validity of the Chinese version of the 17-item HDRS has been investigated and is supported [76].

2) Suicidal ideation: The Scale for Suicidal Ideation (SSI) [77] will be used to assess current suicidal ideation. The Chinese version of the Geriatric Suicide Ideation Scale (GSIS-C), the adaptation of the SSI to be used in the current study, demonstrated excellent internal consistency. In terms of convergent validity, the GSIS-C correlated significantly and positively with depression (assessed by CES-D), loneliness (assessed by Revised UCLA Loneliness Scale), and hopelessness (assessed by Beck Hopelessness Scale) [78].

3) Cognition function: The objectives of this assessment are to provide a measure of cognitive dysfunction and identify cognitive impairments that may associated with depression or/and development of dementia. The Mini-Mental State Exam (MMSE) [79]will be chosen as a commonly used, well validated, rapidly administered global measure of cognitive function. The Chinese version of MMSE showed a sensitivity of 77% and specificity of 70%[80] on diagnoses of dementia.

4) Anxiety: The Clinical Anxiety Scale (CAS) [81] will be used to assess anxiety symptoms. CAS is a 6-item scale derived from the Hamilton Anxiety Scale (HAMA) after deletion of items overlapping with depression. Research supports the reliability and validity of scores derived from the Chinese version of the CAS [82].

5) Medical health: The Cumulative Illness Rating Scale (CIRS)[83], a reliable and validated measure of medical burden that quantifies the amount of pathology in each organ system, will be use to assess subjects' medical health status. In our preliminary studies, we translated and used the CIRS. After the manual training, inter-rater reliability was 0.95.

6) Quality of life: The 12-item Short-Form Health Survey (SF-12) [84] is a commonly used measure of quality of life (QOL). The Chinese version of SF-36 functioned in general population of Hangzhou quite similarly to the original American version, with comparable, and adequate, internal consistency and test-retest reliability. The Cronbach's α coefficients ranged from 0.72 to 0.88 for the social functioning scale and 0.66 for the vitality scale. Two weeks test-retest reliability coefficients ranged from 0.66 to 0.94 [85].

7) Treatment Stigma (TS): To assess patient's stigma regarding medication treatment for depression, three items will be used. The 3 questions which are adapted from Givens' study [86], include: If I were taking a prescribed medication for depression: a) I would feel ashamed (yes/no), b) I would feel comfortable telling my friends or family(yes/no), and c) I would feel okay if people in my community knew(yes/no).

8) Satisfaction: The Client Satisfaction Questionnaire 8-item (CSQ-8) [87,88] is regularly used to assess patient's satisfaction with their service utilization. The psychometric characters of CSQ-8 have been demonstrated and it has been shown to perform as well as the CSQ-18, and often better. The authors report high internal consistency (Cronbach's alpha coefficient > 0.8) and this scale successfully distinguishes degrees of satisfaction even at high levels of satisfaction. We will translate and adapt it specifically for our study. We will assess patients' satisfaction with their treatment in PCCs via the CSQ-PCC.

#### Providers' level

1) Social-demographic: General information including age, gender, education, and martial status will be assessed, and number of years worked in PCCs, which may affect physicians' adherence, will also be assess to be used as a co-variate in all analyses.

2) Clinical practices: The Practice Questionnaire (PQ) will be used to be as a standardized document to measure providers' adherence to treatment guidelines. The PQ includes four questions: a) If clinicians prescribe Sertraline (Yes/No)? b) If clinicians document an evaluation of suicidal risk (Yes/No)? c) If clinicians provide an appointment for follow-up (Yes/No)? d) If clinicians transfer a complicated patients to a mental health specialist (Yes/No)? We will design a Clinical Record Form for the specific rating of patient symptoms, side effects and clinician medication strategy for recording providers' practice. We will also use the medical records (MR) to track providers' clinical practices, including antidepressant prescriptions, documented evaluations of suicide risk, and provision of an appointment for follow-up.

3) Attitudes/knowledge: The Depression Attitude Questionnaire (DAQ) [89] will be administered at baseline to assess PCPs' attitudes/knowledge regarding depression, and administered at the conclusion of the study to quantify changes in attitudes/knowledge after participating in DCM. The DAQ has been used to assess PCPs' attitudes/knowledge towards depression in different countries [90,91]. The author of the DAQ has provided us with the Chinese version of DAQ.

4) Satisfaction: We will assess providers who are in the DCM intervention arm on their satisfaction with the DCM program via the CSQ-DCM. The CSQ-DCM will be adapted from the CSQ and be specific to this study protocol.

#### Other outcomes of interest

The ultimate goal of the intervention is to improve the full range of adverse outcomes associated with depression in later life. While depression symptom severity and associated quality of care indicators are our focus, we also will track psychiatric hospitalizations, incident suicide attempts, and deaths due to suicide and other causes. These outcomes will be too infrequent in a study this size to allow for statistical comparison, but will be useful for descriptive purposes and planning for future, larger scale studies.

### Data management and analysis

#### Data management

Forms will be created for the encoding of data. The forms will be available as hard copy and as computer-based forms to allow direct encoding of data during subject interviews. These data will be stored in the Department of Psychology in Zhejiang University using the web-based communication system (as developed with biostatistics and programmers). Program procedures (as developed with Biostatistics) ensure adequate error checking, security safeguards, and computer backups. Biostatistician collaborators primarily use SPSS for data analysis. Records of subject contacts will be kept by the Program Manager on personal computers using Excel software.

#### Statistical Analysis

The power analysis takes advantage of the longitudinal design for the repeatedly measured outcomes such as depression symptom severity. We assume an average correlation of 0.5 among three follow-up assessments. We further assume a drop-out rate of up to 15% based on prior similar studies. Finally, we adjusted the sample size to accommodate the nesting of patients within clinics (given the patient is the unit of analysis and randomization is at the clinic level). Our experience with nested study data in similar settings show that the intraclass correlation (ICC) has varied from close to 0 to 0.05. Our analyses will assume the higher ICC of 0.05 (lower ICCs will result in greater power). With 16 clinics (8 randomized to CAU and 8 to DCM) and 20 depressed subjects from each clinic, we will have a sample of 320 depressed subjects with 160 per treatment condition.

The sample size of 320 will allow detection of an average between-group effect size of about 0.37 for continuous outcomes over the three follow-up assessments for the repeatedly measured outcomes such as depressive symptoms. Effect sizes for major depression in PCP care ranges from 0.17 to 1.1 based on a meta-analysis study [92]. Thus, the proposed sample is sufficiently powered to detect clinically meaningful changes in this primary outcome of interest.

The sample size will also detect an odds ratio of about 1.8 for the analysis of the binary outcome of remission status; with an estimated remission rate of 0.6 for the intervention group and a squared multiple correlation of 0.3 among the covariates. Similarly, we will be able to detect an odds ratio of 1.7 for the treatment condition in the analysis of the binary outcome of adherence to treatment guidelines with an estimated rate of 0.7 for the intervention group and a squared multiple correlation of 0.3 between the covariates. Odds ratios for adherence for depressed patients in PCP care ranges from 1.3 to 5.6 based on a meta-analysis study [93]. The proposed sample size is sufficient to detect clinically meaningful changes in adherence to treatment guidelines for our study. Note that since data on adherence is collected longitudinally over the assessments and power was computed based on a cross-sectional design, the detectable odds ratio 1.7 is conservative and even a smaller odds ratio can be detected.

#### General Considerations for Data Analysis

Prior to hypotheses testing, we will conduct preliminary analyses to assess whether random assignment of physicians to groups produces groups that are comparable with respect to demographic and baseline clinical conditions. Measures of demographic characteristics and outcomes at baseline will be examined using t- (for continuous variables) or chi-square (for nominal variables) tests. For non-normal continuous variables, nonparametric methods such as Wilcoxon rank sum test will be used. These comparisons will enable the identification of covariates for use in later analyses for comparing group difference. We will also perform descriptive statistics prior to testing the hypotheses. These analyses include means (or frequencies and proportions for categorical variables) and standard deviations. Care Managers' intervention fidelity and implementation will be examined to ensure that the intervention protocol is followed and delivered as intended.

Hypothesis testing will involve comparisons of two groups. This is a longitudinal study where each patient will be measured four times at baseline and every four months post-baseline. Since repeated measures of the same patient are correlated, methods for longitudinal studies will be applied to address the dependence among the repeated outcomes.

The longitudinal design of the study calls for analyses of repeated-measures data. The two most popular approaches for longitudinal data modeling are the weighted generalized estimating equations (WGEE) and generalized linear mixed-effects model (GLMM). Both approaches achieve valid inference under the two popular missing data mechanisms, the missing completely at random (MCAR) and the missing at random (MAR) models. As WGEE does not require any distributional assumption, it provides more robust inference than its counterpart GLMM, WGEE will be used for estimation of intervention effects if discrepancies between GLMM arise.

We will model the mean response by including treatment groups, time, and time by treatment group interaction, controlling for patients' baseline and demographic characteristics if the latter differentiate treatment groups at baseline. A significant time by group interaction will indicate differential changes between the two groups over time. For the GLMM and WGEE analyses, model selection procedures will be applied to find the most parsimonious model using Akaike's information criterion (AIC) as a guide.

#### Analysis of Specific Aims

Aim 1: For primary outcome on patient level outcomes, we hypothesize that patients in DCM practices will have greater reduction in depression symptom severity, and that a larger proportion will achieve clinical remission of depression, than patients who receive CAU.

We will examine changes in depression symptom severity over time between the two treatment conditions using the longitudinal models for continuous outcomes. We will include as explanatory variables in the model the treatment and time main effects plus their interaction, as well as the covariates that significantly predict treatment assignment at baseline. A significant interaction will indicate a significant difference between the two treatment conditions.

Since the remission status is assessed at 16 weeks, the data is cross-sectional. Thus, we will test differences in remission rates between the two treatment conditions using logistic regression. We will include as explanatory variables in the model the treatment condition and the covariates that significantly predict treatment assignment at baseline. Differences in this outcome between the two groups will be indicated by a significant treatment main effect.

Note that we do not expect many subjects with missing data at 16 weeks. In case the number of subjects with missing data is large, we will model the missingness using logistic regression with missing data status as the dependent and treatment and other covariates as explanatory variables. We will then control for the significant explanatory variables from the missing data model as covariates in the logistic regression for remission rates.

Aim 2: For provider level outcomes, we will examine whether DCM are associated with greater adherence to specific quality indicators for depression treatment. We hypothesize that the proportion of depressed subjects who receive an antidepressant prescription, have documented evaluation of suicide risk, and receive an appointment for follow-up within two weeks of prescription will be greater for the DCM group than CAU.

We will test this provider-level (or rather aggregated patient-level) outcomes hypothesis using the longitudinal models for binary outcomes discussed under the General Considerations for Data Analysis. We will include as explanatory variables in the model the treatment and time main effects plus their interaction, as well as the covariates that significantly predict treatment assignment at baseline. A significant interaction will indicate a significant difference between the two treatment conditions.

Aim 3a: For provider level outcomes, we hypothesize that providers in DCM practices will have greater improvements in knowledge/attitudes about depression than those are in CAU practice.

We will use the same modeling strategy for longitudinal data as in Aim 1b to examine this hypothesis. Since the provider level data being very limited given that only a total of 16 clinics will participate in the study, the analysis is largely descriptive, with the model estimates providing information about differential improvements in knowledge/attitudes about depression between the DCM and CAU practices.

Aim 3 b: For patient level outcomes, we hypothesize that patients in DCM practices will have greater improvements in functioning and quality of life, greater reduction of stigma to depression treatment, and greater satisfaction with their care, than patients who receive CAU.

We will use the same modeling strategy for longitudinal data as in Aim 1b to examine this hypothesis. A significant interaction will indicate a significant difference between the two treatment conditions.

### Ethical considerations

For the protection of the subject's privacy and confidentiality, we will use the following steps:

1) During the informed consent process, we will address potential subjects' concerns about protection of their identities against undesired intrusions by the research team and others (privacy) and about limiting access to study information that might identify them (confidentiality).

2) Subjects will be interviewed in a room within the primary clinic or their homes in a manner that assures their privacy. The site of the interview will be the subject's choice.

3) In order to protect the confidentiality of subject information, we will take a number of precautions. These include training of research interviewers in confidentiality procedures; entry and storage of data using coded identification labels; maintenance of hard copy data forms and project computers in locked and secure locations with restricted access by enforced password protection. Back-ups of all electronic study files will be made regularly to allow for recovery of data due to disk failure. We will minimize risks associated with subject burden or distress by employment of research personnel with appropriate backgrounds and experience and work with psychological factors and elderly subjects.

The most common side effects of Sertraline are sleepiness, nervousness, insomnia, dizziness, nausea, tremor, skin rash, and upset stomach, loss of appetite, headache, diarrhea, abnormal ejaculation, dry mouth and weight loss. Important side effects are irregular heartbeats, allergic reactions and activation of mania in patients with bipolar disorder. Most common side effects will disappear in 1-2 weeks without any treatment. In this study, we reduce the start dose as 25 mg/d to avoid most side effects for the concern of age and tolerance. For important side effects, PCPs will report to psychiatrists and let them take care of them.

In this study, patients in CAU group means that they will not receive any intervention from our study team. They consent to be recruited and be assessed with depression and suicide risk. According to the usual care in primary care setting in our study place, most patients with depression have no any treatment or intervention; some PCPs may give them some advice or refer them to other professional institutions, a very small part of patients may visit psychologists or psychiatrists. In our study, all patients will be assessed with suicide risk. The assessment of suicide risk will be based initially on the item i (Thoughts that you would be better off dead or of hurting yourself in some way) of the 9-item Patient Health Questionnaire (PHQ-9). If a patient endorses active or passive suicidal ideation (any answer except "not at all"), the person administering the questionnaire (patient's PCP, nurse or research assessor) will follow a standard DCM Study Safety Protocol (see the supplement). That is, they will be required to ask the following 5 questions and then call the psychiatric team as specified by the Study Safety Protocol to review results an implement interventions as indicated. The psychiatric team will be readily available during the research period. The five questions are:

1) In the past month, have you made any plans or considered a method that you might use to harm yourself? Y/N

2) Have you ever attempted to harm yourself? Y/N

3) There's a difference between having a thought and acting on a thought. Do you think you might actually make an attempt to hurt yourself in the near future? Y/N

4) In the past month have you told anyone that you were going to take your own life, or threatened that you might do it? Y/N

5) Do you think there is any risk that you might hurt yourself before you see your doctor the next time? Y/N

## Discussion

Since most studies focusing on collaborative care have been conducted in the US, an important question is whether or not the positive outcomes of this intervention can be replicated in China. Based on the Chronic Disease Management Model and the core components from the collaborative care model for depression in USA, some CCM trials were conducted for depression in other countries. A randomized controlled trial in the United Kingdom (UK) has shown that the CCM for older people with depression, used in primary care settings, was more effective than usual general practice care and its feasibility and effectiveness were acceptable in the UK [94]. In the Netherlands a trial emphasized factors influencing the CCM implementation and concluded that implementation depended on changes at all levels of the PCC system [95]. The primary care setting is a promising domain for the management of late life depression in China, however, its effectiveness must be tested empirically, and this research needs to be conducted in Chinese primary care settings with Chinese providers and patients.

During our preliminary investigation in Chinese primary care settings, we found that PCPs did not diagnose and treat patients with depression. If they recognized potential cases of depression, PCPs suggested that patients visit mental health specialists. Currently, there is not a transfer mechanism between PCPs and mental health specialists (i.e., direct referrals) and most PCPs are not aware of mental health specialists in their city. Occasionally, PCPs prescribed antidepressants following mental health specialists' protocols on patients' medical records. Based on these preliminary data we propose that a system-wide intervention is needed in PCCs that will involve the collaboration of PCPs, nurses as care managers, and psychiatrists as mental health specialists, as well as the promotion of treatment guideline-driven care, as a collaborative care management intervention for depressed, older patients.

Thus, our model includes PCPs, nurses, psychiatrists, treatment guidelines, psychiatric consultation and communication as the Depression Care Management (DCM) model for late life depression in Chinese primary care settings. As the first randomized controlled trial of CCM for late life depression in China, we acknowledge that the trial intervention will not tackle all the barriers to the integration of depression management in primary care, and the medication treatment is not well enough for the needs of patients. How to design an effective and evidence-based psychotherapy for the management of depression, how to integrate other useful resource into the model, how to use this model in big rural area in China, these question will be addressed in future research. The proposed trial will provide the first systematic evidence for the collaborative care model for mental health problems in late life, and will be helpful for the government's decision-making on the supporting mental health program in China primary care settings.

## Abbreviations

DCM: depression care management; CAU: care as usual; PCPs: primary care physicians; SSRIs: selective serotonin reuptake inhibitors; STAGED: Somatic Treatment Algorithm for Geriatric Depression; PCC: primary care clinic; TG: treatment guideline; CCM: collaborative care model; CM: care manager; CRC: clinical research coordinator

## Competing interests

The authors declare that they have no competing interests.

## Authors' contributions

SC, MD was responsible for the initial drafting of the paper and for coordinating the revisions leading to the final paper for submission. YC, MD provided monitoring and guidance on this paper. BX, PhD and XT PhD provided specific inputs for the statistics section of the paper. MY, MD provided the inputs for the health policies for primary care, and the research sites and environment section of the paper. All authors have read the paper and are in agreement with the contents.
